# Updated Genome Assembly of Bighead Carp (*Hypophthalmichthys nobilis*) and Its Differences Between Male and Female on Genomic, Transcriptomic, and Methylation Level

**DOI:** 10.3389/fgene.2021.728177

**Published:** 2021-09-06

**Authors:** Beide Fu, Ying Zhou, Haiyang Liu, Xiaomu Yu, Jingou Tong

**Affiliations:** ^1^State Key Laboratory of Freshwater Ecology and Biotechnology, Institute of Hydrobiology, The Innovation Academy of Seed Design, Chinese Academy of Sciences, Wuhan, China; ^2^University of Chinese Academy of Sciences, Beijing, China; ^3^Key Laboratory of Tropical and Subtropical Fishery Resources Application and Cultivation, Ministry of Agriculture and Rural Affairs, Pearl River Fisheries Research Institute, Chinese Academy of Fishery Sciences, Guangzhou, China

**Keywords:** updated, sex determination, chromosomal assembly, bighead carp, male-specific region

## Abstract

Cyprinidae is one of the largest family in freshwater fishes, and it is most intensively cultured fish taxon of the world. However, studies about sex determination in this large family is still rear, and one of the reasons is lack of high quality and complete genome. Here, we used nanopore to sequence the genome of a male bighead carp, obtaining contig N50 = 24.25 Mb, which is one of the best assemblies in Cyprinidae. Five males and five females were re-sequenced, and a male-specific region on LG19 was confirmed. We find this region holds many male-specific markers in other Cyprinidae fishes, such as grass carp and silver carp. Transcriptome analyses of hypothalamus and pituitary tissues showed that several sex-specific differentially expressed genes were associated with steroid biosynthesis. The *UCH64E* gene, located in the male-specific region on LG19, showed higher expression levels in male than female tissues of bighead carp. The methyl-RAD of hypothalamus tissues between males and females indicated that the sexual methylation differences are significant in bighead carp. We also compared the methylation sites recognized using methyl-RAD and nanopore raw reads and found that approximately 73% of the methylation sites identified using methyl-RAD were within nanopore CpG sites.

## Introduction

The mechanism about sex determination in fishes is astonishing variable and have occurred independently and repeatedly (Bachtrog et al., 2014; Furman et al., 2020). Both genetic and environmental factors can control sex-determination system in fishes, but only a small perentage of teleost fish have been proved to have sex chromosome (Kottler and Schartl, 2018). For many other fishes with genetic sex determination (GSD), they only have sex-specific regions in their genome. Many studies have proved that sex determination genes (STDs) were located in sex-specific region, such as in Atlantic herring (Rafati et al., 2020) and medaka (Kondo et al., 2006; Myosho et al., 2012). Besides the genomic difference between male and female, other studies also found sex-specific expressed genes in different fishes, such as in half-smooth tongue sole (Lin et al., 2020), olive flounder (Ryu et al., 2020), and clownfish (Casas et al., 2016). With the development of sequencing technology for epigenetics, methylation difference between male and female fishes were also found. For example, methylation may abrogate the stimulation of *cyp19a1a* by gonadotropins in a male-specific fashion in ricefield eel (Zhang et al., 2013). Zhou et al. (2019) also found whole genome methylation difference for sex changes under low temperature in *Takifugu rubripes*. Above all, sex-determination system in fish is complicated and needs different level of data to uncover the key modules which played key roles in it (Ortega-Recalde et al., 2020).

With more than 1,600 species distributed in different continents, Cyprinidae is one of the largest family of freshwater fishes in the world (Nelson, 2006). But sex determination studies have been conducted only in few species of this large group (Zhang et al., 2017; Liu et al., 2018). As a model experimental species in Cyprinidae, wild zebrafish has been found to have ZZ/ZX sex determination (SD) system and after human selection the strain in labs lost their GSD system (Kossack and Draper, 2019). However, previous studies have shown that many fishes use XX/XY sex determination system in this large group (Zhang et al., 2017; Feng et al., 2018; Liu et al., 2018; Zhou et al., 2020), but these studies are far away from understanding the sex-determination system, which needs the help from genomic, transcriptomic and epigenetic data.

High quality genome sequence is a prerequisite for sex-determination studies in both model and non-model species. Bighead carp (*Hypophthalmichthys nobilis*, NCBI Taxonomy ID: 7965) is one of representative Cyprinidae species in East Asia, and its first version genome has been sequenced by Wang et al. (2020a). The assembled genome from a male bighead carp was not sorted into chromosomes and highly fragmented (N50 = 83 kb). Then, Jian et al. (2020) used high density genetic map to anchor scaffolds to 24 chromosomes for bighead carp, but the updated version is still lack of high continuity.

In this study, we used nanopore sequencing and Hi-C scaffolding to assemble a chromosomal-level, male bighead carp genome. Genomic comparison between male and female bighead carps was carried out to identify male-specific regions based on the genome, with excellent continuity at the scaffold level. The genome of male bighead carp may provide solid insights and valuable genomic data for further studies on sex-determination mechanisms, genetic paradox after invasion, and marker-assisted breeding of bighead carp.

## Materials and Methods

### Fish Sample and Sequencing

All experimental procedures in this study were approved by the Committee of Animal Care and Use at the Institute of Hydrobiology, Chinese Academy of Sciences. A 1-year old male bighead carp was used as the sequencing sample. The parents of the sample fish were collected from the Yangtze River and reared in Zhangdu Lake Fish Farm in Wuhan, China (GPS location: 30°40′06.12 N, 114°44′26.34 E). High-quality genomic DNA was extracted from muscle tissue using Blood & Cell Culture DNA Midi kit (Q13343, Qiagen, CA, United States). We chose muscle tissue because ultralong nanopore sequencing needs high-quality genomic DNA and it can be easily got from fresh muscle tissue. The quality and quantity of DNA were assessed using Agilent Bioanalyzer 2100 (Agilent Technologies) and Qubit double-stranded DNA HS Assay Kit (Invitrogen, Thermo Fisher Scientific). For nanopore sequencing, the library was constructed according to the manufacturer’s instructions and sequenced on R9.4 Flow Cell using PromethION at Biomarker Technologies Corporation (Beijing, China).

To obtain a chromosome-level assembly for the bighead carp genome, a Hi-C library was constructed using the standard procedure with muscle tissue from the same sample as that for nanopore sequencing. First, fresh genomic DNA from the muscle tissue was cross-linked using formaldehyde *in situ*. Second, the *Hin*dIII enzyme (NEB) was used to digest the cross-linked DNA, and sticky ends were marked with biotin. Third, the biotin-labeled DNA was enriched and unlinked to shear to a size of 300–700 bp (Lieberman-Aiden et al., 2009). Finally, a 150-bp pair-end library with an insert size of 350 bp was constructed according to Illumina protocol and sequenced on the Illumina HiSeq platform at Biomarker Technologies Corporation.

To identify sex-specific regions in the male bighead carp genome, the DNA of muscle tissues from five male and five female 1-year-old bighead carp was sequenced on the Illumina NovaSeq 6000 platform at Biomarker Technologies Corporation. Filtered reads were mapped to assembled female genome with Bowtie2 version 2.3.5.1 (Langmead and Salzberg, 2012) and only high quality mapping results were kept (mapping quality over 20). Then coverage for each 50 kb block were calculated by Samtools v1.10 (Li et al., 2009). Then, *t*-test were calculated in R v3.4.0^1^ to find regions with significant difference on coverage for all males and females.

### Genome Assembly

First, a 17-bp k-mer frequency distribution was used to estimate genome size (Li et al., 2010). NextDenovo v2.3^2^ was used to assemble filtered nanopore reads to the contig level using default parameters. Illumina reads from one male bighead carp were mapped to the assembly using Bowtie 2 (Langmead and Salzberg, 2012). Pilon v1.22 was used twice with a bam file in the last step to correct errors in assembly (Walker et al., 2014). The corrected assembly was anchored to the chromosome using LACHESIS (Burton et al., 2013). BUSCO v3.0.2 was used to assess the completeness of the chromosome-level genome with actinopterygii_odb9 and -m geno -sp zebrafish settings (Waterhouse et al., 2018).

### Annotation of Repeative Elements and Genes

Repetitive elements were identified using both *de novo* and homology-based methods. First, *de novo* repeat annotation within the bighead carp genome was carried out using RepeatModeler v1.0.11^3^ with default parameters. Second, the repetitive elements found by RepeatModeler were combined with Teleostei repeats from RepBase-20181026 and Dfam_Consensus-20181026 databases. The combined library was used in further homology-based transposable element searching at the DNA level using RepeatMasker v4.0.8^4^ with -nolow -no_is -norna -engine NCBI parameters. Telomere sequences were identified by searching among the RepeatMasker outputs for high copy number with the repeat pattern TTAGGG.

The gene model was predicted by incorporating *ab initio* prediction, homology-based prediction, and RNA-Seq assisted prediction. The *ab initio* prediction was carried out using AUGUSTUS v3.3.2 with species = zebrafish parameter (Stanke et al., 2006). The protein sequences used for homology-based prediction were obtained from zebrafish, medaka, fugu, and tilapia in the Ensembl database (Flicek et al., 2011). RNA-seq data from the brain, liver, muscle, and gill obtained by Illumina sequencing were used for RNA-seq assisted prediction. Finally, all three results were combined using EVidenceModeler v1.1.1 (Haas et al., 2008). Comparative genomic synteny analysis between bighead carp and zebrafish was carried out using MCScanX (Wang et al., 2012).

### Transcriptome Analysis

To compare transcriptomic differences between male and female samples, we sequenced the hypothalamus and pituitary transcriptomes of two male and two female bighead carp. As bighead carp sex mature at 3- or 4-year-old, gonad tissue of it is morphological distinguishable easy to be collected at 2.5-year-old. The four samples were 2.5 years old, and they were collected from Shishou, China. The sex was determined by the shape of their glands and further verified by a male-specific marker. In addition, testis tissues from the two male bighead carps were collected and sequenced on the Illumina platform. The mRNA from these tissues was extracted using a Promega Z3100 kit (Promega, United States), and quality was checked using NanoDrop 2000. Sequencing was carried out using Illumina HiSeq 2500, and the raw reads were quality-filtered using FastaQC v0.11.3.^5^ Filtered reads were mapped to the assembled genome using HISAT v2.1.0 with default parameters (Kim et al., 2015). Gene quantification was carried out using HTSeq2 (Anders et al., 2015), and differential expression analysis was performed using DESeq2 (Love et al., 2014). Differentially expressed genes (DEGs) were maintained with fold-change > 2 and adjusted P value (FDR) < 0.01.

### Methylation Analysis for Nanopore Reads

The methylation level of 5mC in the muscle tissue of male bighead carps was investigated using Nanopolish (Simpson et al., 2017). We mapped raw nanopore reads to the assembled genome using minimap2 (Li, 2018) and sorted them using SAMtools v1.8 (Li et al., 2009). We removed any position with less than 10 reads that could cause false positives. Finally, we calculated the methylation frequency and log-likelihood ratio at each position.

### Differential Methylation Analysis Between Male and Female Bighead Carp

Genomic DNA from the hypothalamus tissue of three male and three female bighead carps was used for methyl-RAD library construction as described by Wang et al. (2015). The six libraries with equal amounts of DNA were mixed and sequenced on an Illumina HiSeq 4000 platform with paired-end mode. After obtaining the raw sequencing data, reads with ambiguous base calls (N) or excessive numbers of low-quality bases (sequencing quality lower than 10) were removed. High-quality reads were mapped to the assembled genome using HISAT2 (Kim et al., 2015), and quantification was carried out using Python scripts. Methylation levels were determined using the qCML method implemented in the edgeR package in R v3.6.1 (Robinson et al., 2010).

## Results

### Nanopore Sequencing and Genome Assembly

**FIGURE 1:**
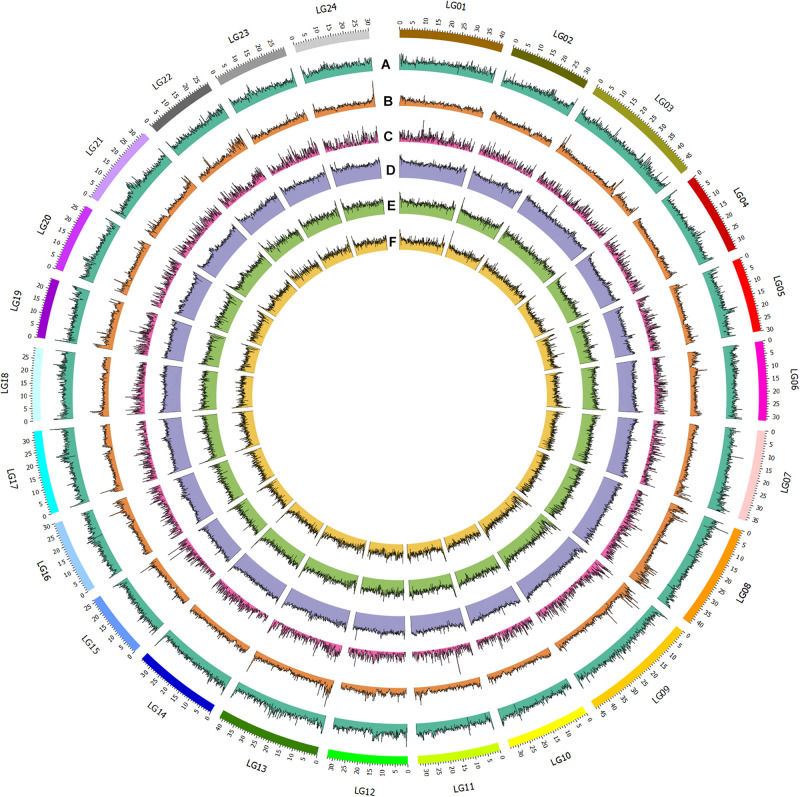
Genomic and epigenomic feature of bighead carp. From outer to inner circle: A. GC content; B. SNP distribution (100kb window); C. Gene distribution; D. Nanopore methylation sites; E. MspJI methylation sites; F. FspEI methylation sites.

In this study, we used ultra-long nanopore reads to obtain the whole genome of a male bighead carp and obtained 79.82 Gb of raw data. After filtering adaptors, short reads, and low-quality reads, 69.59 Gb of clean reads were retained for further assembly, which corresponded to approximately 78.83 × coverage. The longest read was 417,624 bp in size, and the mean quality value of all bases was 8.33 (Supplementary Table 1). The average length for filtered reads was 28.84 kb, and over 82% of them were longer than 10 kb, accounting for more than 96% of all bases (Supplementary Table 2 and Figure 1). These high-quality reads were assembled and polished in a stepwise manner to the contig level, and the generated 857.04 Mb genome size had contig N50 of 24.25 Mb. This genome contained only 77 contigs, and the longest one was 49.7 Mb. A total of 57 contigs were longer than 1 Mb, covering 99.14% of the genome. The GC content of the contig-level genome was 37.33%.

**FIGURE 2:**
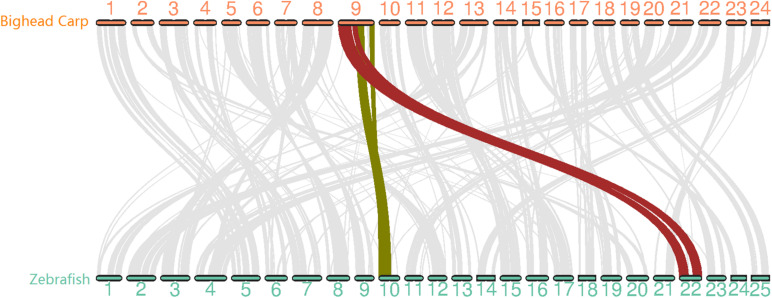
Whole genome synteny analysis between bighead carp and zebrafish genome with 12,072 gene pairs. The chromosomes in the two genomes showed 1-to-1 relation except for LG9 in bighead carp corresponding to Chr 10 and Chr 22 in zebrafish.

**FIGURE 3:**
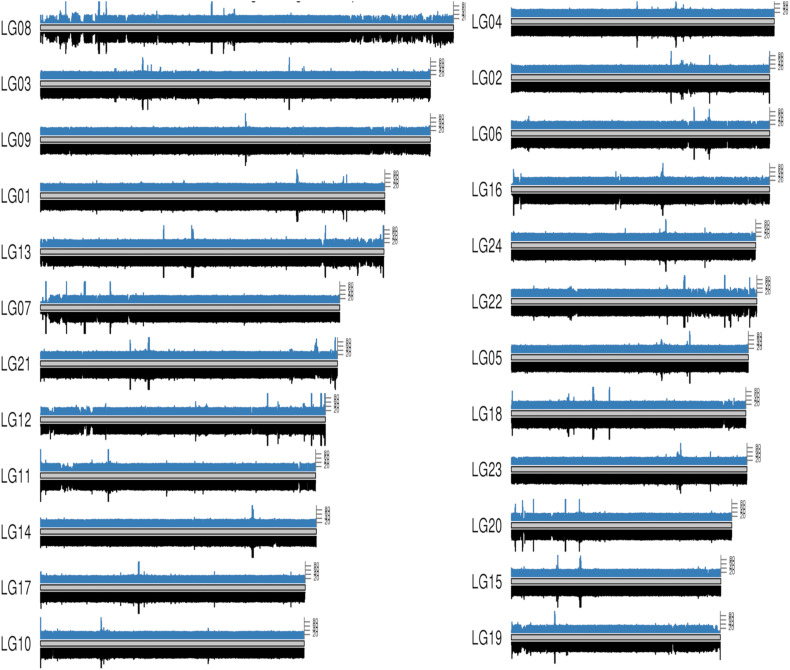
Whole genome coverage on male and female bighead carp genome. The blue bars on each chromosome come from a female bighead carp and the black bar below come from a male. Each bar represents a 50 kb region on genome.

To assemble the contig-level genome to chromosomes, the Illumina X Ten platform was used to sequence a muscle Hi-C library. Then, 97.64-Gb Hi-C data were generated, 84.57% of which were valid interaction pairs and used to anchor 856.54 Mb of contigs (99.94%) to 24 chromosome-level scaffolds (Supplementary Table 3 and Figure 2). Finally, we obtained an 857.04-Mb bighead carp genome comprised of 32 sequences, accounting for 96.83% of the estimated genome size (884.62 Mb, Supplementary Table 4). The lengths of 24 chromosomes of the bighead carp genome ranged from 26.63 Mb (LG19) to 52.63 Mb (LG08), with an average size of 35.66 Mb (Figure 1 and Supplementary Table 5 and Figure 3). The scaffold N50 for the final assembly was 33.74 Mb, which was 406.50-fold and 10.13-fold higher than those described by Wang et al. (2020a) and Jian et al. (2020), respectively. In addition, only 5.5 kb of gaps were present in our assembly, a 13,220.71-fold and 2,076.55-fold reduction compared to the previous bighead carp genome (Table 1; Jian et al., 2020; Wang et al., 2020a).

**TABLE 1:** Genomic statics for our assembly and previously published two bighead carp genome.

	Wang et al.	Jian et al.	Our assembly
Contig Statistics			
Number of contigs	742,098	175,081	77
Total contig size (bp)	N.A.	844.66 Mb	857.04 Mb
Contig N50 size	4051 bp	39.1 kb	24.25 Mb
Largest contig (bp)	N.A.	376623bp	49.70Mb
Scaffold Statistics			
Number of scaffolds	121,326	121,449	32
Total scaffold size	1.08 Gb	845.5 Mb	856.54 Mb
scaffold N50 size	83 188 bp	3.33 Mb	33.74 Mb
Largest scaffold	673,715 bp	19.31 Mb	52.63 Mb
Gap length	72,713,928	11,421,027 bp	5500 bp
GC content (%)	37.38	37.2	37.33
Repeat elements ratio (%)	43.50	45.04	50.29
Genes found in BUSCO (%)	N.A.	96.2	95.2

**FIGURE 4:**
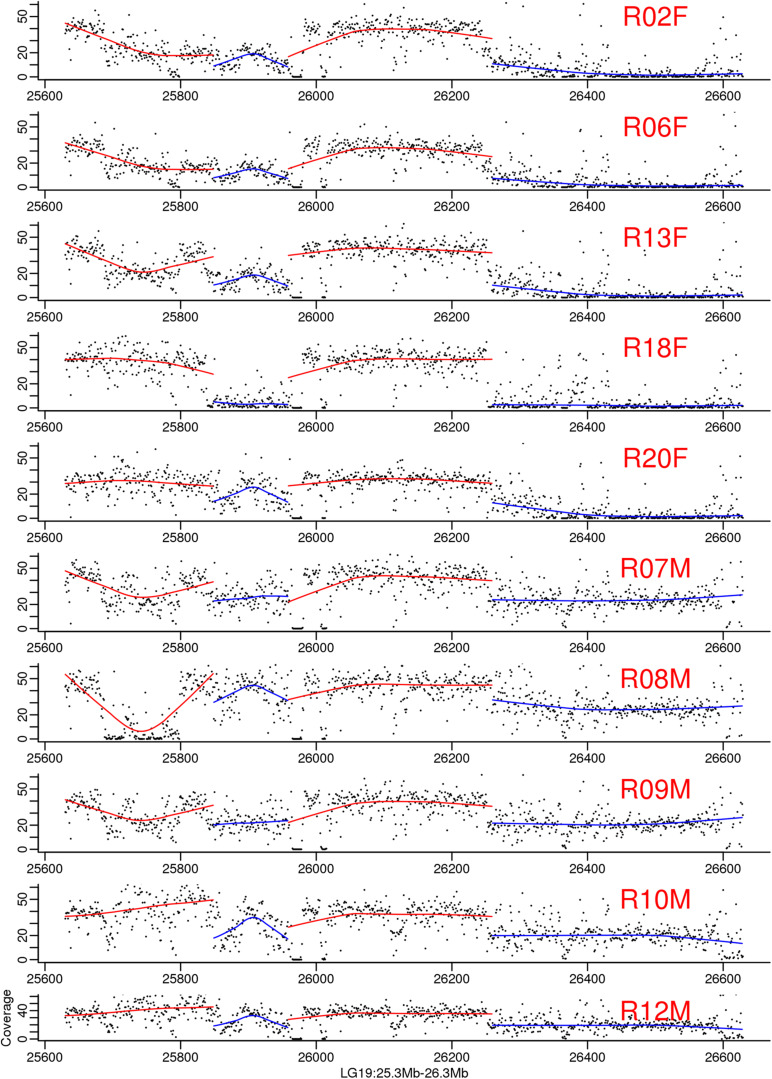
Genomic coverage on the largest male-specific region of bighead carp genome. The two blue regions are male-specific region on LG19. Each dot in the figure represented a 1 kb region. The line is regression for each part. The samples ended with F means they are female and whose ended with M means they are male bighead carp.

To evaluate the quality of the bighead carp genome, we first searched for telomere sequences and found the presence of telomeres in 35 out of 48 ends, suggesting that our assembly was almost end-to-end sequencing of the bighead carp genome (Supplementary Table 6 and Figure 4). We then mapped 94 Gb of Illumina reads used in polishing the genome assembly and obtained a 97.49% overall alignment rate with an expected insert size distribution, which indicated a high confidence of genome assembly (Supplementary Table 7). We then used BUSCO to evaluate the sensitivity and completeness of genes involved in genome assembly. Our assembly contained 95.2% of the 4,584 core genes in actinopterygii_odb9 (Supplementary Table 8). To carry out whole-chromosome-level synteny comparison between bighead carp and its nearest model species, zebrafish, the longest protein for 17,550 genes in these two species was used in MCScanX (Figure 2). The synteny result was consistent with that described in previous reports, that is, LG9 in bighead carp corresponded to LG10 and LG22 in zebrafish (Fu et al., 2016). In this assembly, we found that the heterozygosity for bighead carp was 0.00355, which was much higher than that detected in the USA (0.0021) (Wang et al., 2020a).

### Annotation

Based on *de novo* prediction and homology search performed using Repbase, we found that 50.28% of the bighead carp genome was composed of repetitive elements (Supplementary Table 9). This ratio was comparable to that of the zebrafish genome (52.2%) and was much higher than the average of other sequenced teleost fishes (approximately 30%) (Kettleborough et al., 2013). Among these repetitive elements, interspersed repetitive elements accounted for 48.33% of male bighead carp genome, including 32.99% of DNA elements, 6.28% of LTR (Long Terminal Repeat) elements, 2.25% of LINEs, and 6.45% of unclassified repeats (Supplementary Table 9). The repetitive elements and their classification in bighead carp is similar to that in zebrafish, in which type II transposable elements are much higher than type I transposable elements (Howe et al., 2013). When compared with the ratio of repeats in previous bighead carp genomes, we found our results to be higher (43.5%), indicating a high-quality performance for the *de novo* genome assembly, with highly repetitive sequences by ultra-long nanopore reads (Wang et al., 2020a). We also annotated 23,656 protein-coding genes in the bighead carp genome, which were similar to previous annotation (Jian et al., 2020). In these genes, we found that 96.4% showed positive results in the SwissProt database.

### Male Specific Region in Bighead Carp Genome

To map the sex determination region (SDR) in the male bighead carp genome, we performed additional WGS sequencing for five male and five female samples, with an average coverage of ∼40 × per individual. We did not find any sex chromosomes between male and female samples (Figure 3). Given that bighead carp have an XY/XX sex determination system, we wanted to find a male-specific region in our genome assembly. We used a stringent method to identify genomic regions whose coverage differed significantly between male and female groups. In total, we found six regions longer than 10 kb in chromosomes or contigs that were specific to male samples, and most of them were located on LG19 and ctg000540_ERROPOS22028501 (Table 2). After checking the HiC anchoring result, we found that ctg000540_ERROPOS22028501 was part of ctg000540, adjacent to the largest sex-specific region on LG19. To further determine the genomic difference between sexes on LG19, we used stringent statistical methods to verify the authenticity of these regions (Figure 4). We found that the male-specific region was divided into two parts by a non-sex-specific region, and the average coverage differed significantly in male-specific regions. In addition, the male-specific region on LG19 contains male-specific markers and markers located in sex-related QTL in bighead carp (Liu et al., 2018; Zhou et al., 2020).

**TABLE 2:** Sex-specific regions on each chromosome or contig in bighead carp genome.

LG name	Count on sex-specific region (kb)
LG19	325
ctg000540_ERROPOS22028501	41
LG08	26
LG11	19
LG18	12
LG12	10
LG16	10

To verify the authenticity of these male-specific regions, we designed primers and verified whether they showed differences between male and female samples (Supplementary Figure 5). The PCR amplification results showed that these regions were reliable when verified on samples from different locations, which had the same pattern as we previously reported (Liu et al., 2018).

### Comparative Transcriptome Analysis Between Male and Female Bighead Carp

**FIGURE 5:**
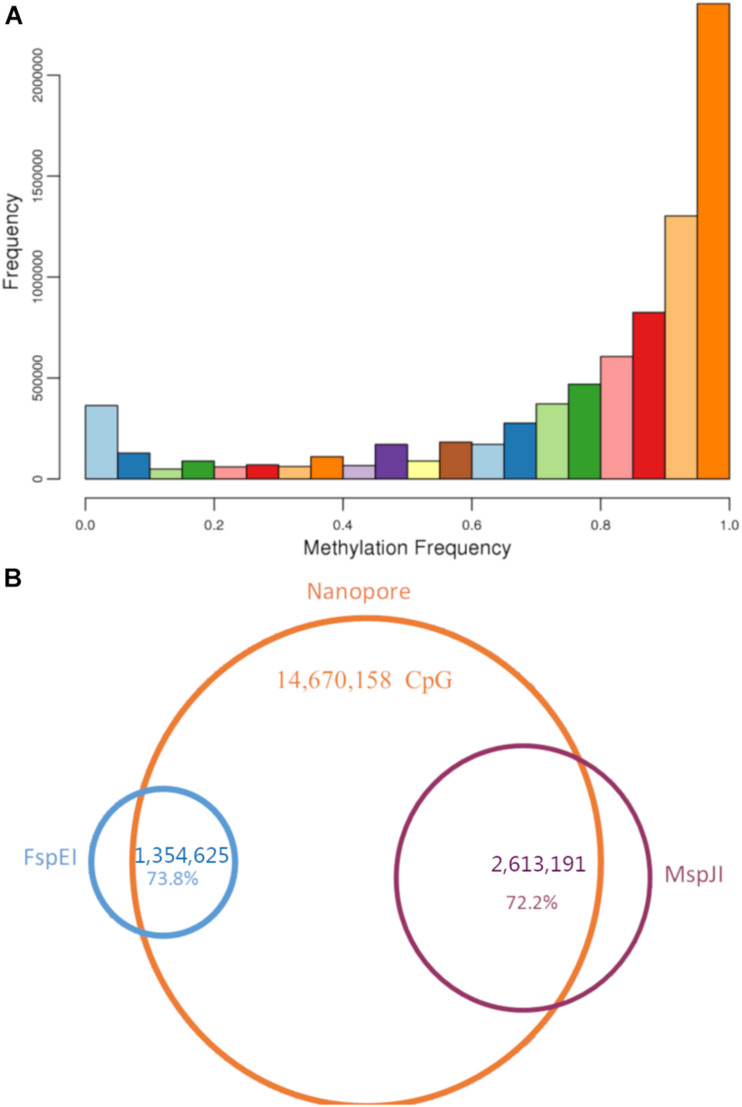
Methylation sites in bighead carp. **(A)** Methylation frequency for all CpG sites in Nanopore raw reads. **(B)** Venn graph for CpG sites in both Methyl-RAD and Nanopore reads. About 73% of CpG sites identified from Methyl-RAD presented in Nanopore reads, too.

To determine the expression pattern between males and females, we collected hypothalamus and pituitary tissues from both sexes of bighead carp during a period of two and a half years. In the hypothalamus, we obtained 9,467 genes left after filtering genes with low or no expression signals (Supplementary Table 10). Among them, 262 genes were differentially expressed, and they were enriched in the insulin signaling pathway (dre04910) and FoxO signaling pathway (dre04069) and in steroid biosynthesis (dre00100) (Supplementary Table 11 and Figure 5). In the pituitary tissue, 8,346 genes were left after removing low-expressed genes, and 234 were differentially expressed (Supplementary Tables 12, 13). These genes were enriched in steroid biosynthesis (dre00100) and glycerolipid metabolism (dre00561) (Supplementary Figure 7). Finally, we identified 74 genes that were differentially expressed in both tissues (Supplementary Table 14). Among these genes, four of the five genes located on LG19 showed BLAST results in the zebrafish proteome, but these target genes have not been well investigated yet. The last one without any hits in zebrafish was searched against the NCBI NR database, and its best hit was ubiquitin carboxyl-terminal hydrolase 64E (*UCH64E*) from *Anabarilius grahami*. We also found that this gene was located in a male-specific region on LG19.

To further utilize the advantage of the full-length transcriptome, we sequenced a brain ONT full-length transcriptome from a male bighead carp. With the help of this full-length transcriptome, we found 2,387 out of 2,442 previously reported lncRNAs in our annotation (Fu et al., 2019). Besides, we found an unreported lncRNA (NCBI accession number: MT786702), with 12 exons.And there are 110 full-length of this lncRNA in the library. This gene was also located in the male-specific region on LG19 (Supplementary Figure 9).

### Methylation in Male and Female Bighead Carp Hypothalamus

To investigate the methylation difference in male and female bighead carps, we used methyl-RAD methods to identify differential methylation sites (DMS) in hypothalamus tissue. In this study, we used both FspEI and MspJI enzymes to obtain the methylation site in 10-month-old bighead carps. For the FspEI enzyme, we obtained 1,354,625 tags containing methylation sites, among which 863,548 were present in more than one sample. After removing tags with too few reads, we found that 544 CpGs were significant DMS between male and female samples (FDR < 0.01). For the MspJI enzyme, we obtained 2,613,191 methylation tags with enzyme digestion sites, and 1,063,504 of them were present in only one library. After removing tags with too few reads, we found that 572 CpGs were significantly differentially methylated between male and female samples (FDR < 0.01) (Supplementary Table 15). Of these 572 CpGs, 68 were annotated genes (Supplementary Table 15). We found that *prkcb*a was within the GnRH pathway, playing a key role in sex differentiation in mammals and fishes (Franco and Yao, 2012; Feng et al., 2020).

In addition, we analyzed the whole genome methylation status of 5mC at CpG with the original fast5 file produced using nanopore sequencing. The methylation frequency (i.e., the proportion of reads supporting 5mC compared with all reads in each CpG) was calculated for each CpG site. In total, we found 14,670,158 CpG sites in the bighead carp genome, and more than 76% of them (11,249,063) had over 10 read support (Supplementary Table 16). In the methylation frequency analysis, we found that more than half of CpG sites in the genome were methylated (Figure 5A). To investigate the overlap of methylation sites between the two methods, we mapped the sites found in methylRAD to nanopore sites and found that 999,713 out of 1,354,625 (73.8%) were present in the FspEI library, whereas 1,886,723 out of 2,613,191 (72.2%) were present in the MspJI library (Figure 5B).

## Discussion

*De novo* sequencing and assembly of highly repetitive teleostei genomes have been challenging and problematic because of the high proportion of repetitive elements. Here, we constructed a high-quality reference genome for bighead carp with ultra-long nanopore reads, which could span the entire length of most repeat units. We assembled an 857.04-Mb *de novo* genome for *H. nobilis*, highlighting the potential of combining nanopore sequencing and Hi-C scaffolding compared to fragmented, hybrid genome assembly with Illumina and PacBio sequencing data (Jian et al., 2020; Wang et al., 2020a). With the help of ultra-long reads generated by nanopore sequencing, we were able to resolve ambiguous regions in the genome caused by long repetitive elements such as the most abundant DNA element.

Although medium- and high-density genetic maps have been constructed for bighead carp in the past several years (Zhu et al., 2014; Fu et al., 2016; Liu et al., 2016), the density of these maps is still far from satisfactory for scaffolding 24 chromosomes with assembled contigs. Therefore, we used long-range Hi-C reads to scaffold contigs assembled from nanopore reads into 24 pseudo-chromosomes, obtaining a considerable improvement in sequence contiguity and a great reduction in gap regions. The scaffold N50 for our assembly is more than 10-fold higher than previous while gap length is over 2,000-fold reduction compared with previous published bighead carp genome (Jian et al., 2020; Wang et al., 2020a). In addition, the telomere repeats found in our assembly indicated that the assembly reached 35 of 48 chromosome ends. In addition, the high proportion of BUSCO evaluations in our improved genome supports the completeness of our assembly.

With the development of high-throughput sequencing, resequencing of male and female samples has become increasingly popular in studies on animal sex-determination systems. Liu et al. (2020) used resequencing to construct a high-density genetic map and QTL mapping of sex traits in pearl oysters. Zhang et al. (2017) used a pool-and-sequence method to identify six Y-linked scaffolds in the grass carp genome and verified it in the wild population. In this study, we identified six male-specific regions longer than 10 kb in the bighead carp genome. The largest one covered over 480 kb on LG19 and harbored one previously reported male-specific marker and three SNP markers located in sex-related QTL (Liu et al., 2018; Zhou et al., 2020). As bighead carp and grass carp belong to the same family, we BLASTed six verified sequences in ctg971, the only verified Y-linked scaffold in the grass carp genome, to the male-specific regions in bighead carp and found that four out of six mapped to the largest region on chromosome 19 (Figure 5; Zhang et al., 2017). These results further support the common origin of sex-specific regions in the diploid endemic clade of East Asia Cyprinidae (Liu et al., 2018).

**FIGURE 6:**
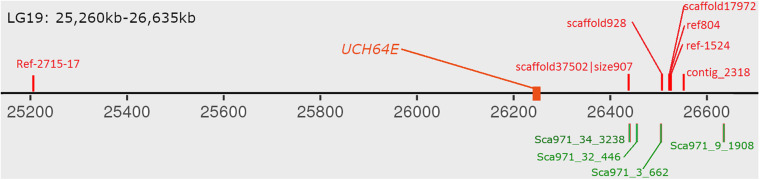
Distribution of verified male-specific markers on male-specific region. The red markers above chromosome are from bighead carp and green markers below are grass carp male-specific markers. *UCH64E* gene are differential expressed gene in both hypothalamus and pituitary tissues.

In some GSD species, STDs were found in sex-specific regions or sex chromosomes such as *sdY* on male-specific regions in rainbow trout (Yano et al., 2012), *dmrt1* on the Z chromosome of half-tongue sole (Chen et al., 2014) and *amhy* on the Y chromosome of tilapia (Li et al., 2015). However, in some species, no STD was found in sex-specific regions with dozens of sex-specific markers (Suda et al., 2019). In this study, we found one gene (*UCH64E*) within the male-specific region that showed distinct expression patterns between male and female tissues (Figure 6). This gene is a candidate sex-determination gene in bighead carp. We further investigated whether this gene originated from other genes in bighead carp, but we did not find another homolog. This pattern is completely different from that in Atlantic herring, which has a sex-determination gene originating from a gene that exists in both male and female individuals (Rafati et al., 2020).

Transcriptomic analysis between different sexes has been carried out in many aquaculture species to identify sex-specific markers and understand sex differentiation processes and sexual development (Ding et al., 2020; Lobo et al., 2020; Shen et al., 2020; Wang et al., 2020b). In this study, we compared the expression patterns in male and female hypothalamus and pituitary tissues. The tissues were collected when the fishes were 2.5 years old, and many eggs were present in the ovaries of bighead carp. Our results showed that many genes were enriched in steroid and glycerolipid biosynthesis, key pathways regulating the synthesis of yolk in eggs. In addition, we found 19 genes located on LG19 that were differentially expressed in the hypothalamus of males and females, among which three genes were located in male-specific regions. These three genes were UCL64E, aquaporin-9-like, and one uncharacterized protein, LOC113042953. Aquaporin-9 (AQP9) has been found to be associated with the amnion epithelium and cytotrophoblast of the chorion (Beall et al., 2012). In the bighead carp hypothalamus, we found that the expression of this gene in females was almost three times higher than in males, which may be associated with pelagic eggs needing water influx after release (Tao et al., 2010).

DNA methylation is a common mechanism of epigenetic regulation in both plants and animals (Jones, 2012). Although gender-specific epigenetic studies have been carried out in *Populus* (Brautigam et al., 2017), humans (Nino et al., 2018), and half-tongue sole (Chen et al., 2018), the function of this important regulatory mechanism in sex determination and differentiation is still unclear. In most studies, whole-genome methylation analysis is carried out using whole-genome bisulfite sequencing (WGBS). However, in this study, we used the original electrical signal generated by nanopore sequencing, which does not require bisulfite conversion or PCR amplification. Therefore, the methylation status identified by nanopore is more sensitive and accurate than WGBS. To our knowledge, this is the first whole-genome methylation study of aquaculture species carried out using nanopore sequencing. We also used methylation-sensitive enzymes to determine the methylation pattern differences between male and female hypothalamus tissues. Most methylated CpGs identified by FspEI and MspJI were present in sites detected by nanopore sequencing. The remaining proportion (∼25%) might be not detected using nanopore sequencing due to tissue-specific difference between the muscle and hypothalamus.

Above all, high-quality bighead carp genome provided a solid fundamental for the following comparative genomics between male and female samples. And the transcriptome data from different gonads further validate the male-specific genes’ expression in male-specific regions. Whole genome methylation pattern in different tissues also shed some light on deciphering the sex determination and sex differentiation in bighead carp. We hope multiple omics data’s combination will be a powerful tool genetics study in future.

## Data Availability Statement

The datasets presented in this study can be found in online repositories. The names of the repository/repositories and accession number(s) can be found below: https://www.ncbi.nlm.nih.gov/, PRJNA657698.

## Ethics Statement

The animal study was reviewed and approved by Committee of Animal Care and Use at the Institute of Hydrobiology, Chinese Academy of Sciences.

## Author Contributions

JT and BF conceived the whole project. XY and YZ planned the selection of samples and data collection. YZ did the methylation library construction. BF assembled and annotated the genome. BF and HL did the analysis between male and female bighead carp. BF and JT wrote the manuscript and all the authors participated in the discussion. All authors approved the final manuscript.

## Conflict of Interest

The authors declare that the research was conducted in the absence of any commercial or financial relationships that could be construed as a potential conflict of interest.

## Publisher’s Note

All claims expressed in this article are solely those of the authors and do not necessarily represent those of their affiliated organizations, or those of the publisher, the editors and the reviewers. Any product that may be evaluated in this article, or claim that may be made by its manufacturer, is not guaranteed or endorsed by the publisher.

## Footnotes

1
https://www.R-project.org

2
https://github.com/Nextomics/NextDenovo

3
https://github.com/rmhubley/RepeatModeler

4
http://repeatmasker.org

5
http://www.bioinformatics.babraham.ac.uk/projects/fastqc/
