# Supplementary material for: Updated Genome Assembly of Bighead Carp (Hypophthalmichthys nobilis) and Its Differences Between Male and Female on Genomic, Transcriptomic, and Methylation Level
Source: Front Genet. 2021 Sep 6;12:728177. doi: 10.3389/fgene.2021.728177 (PMC8452039; doi:10.3389/fgene.2021.728177)
Supplement: Supplementary file 1 [file Image_1.pdf]

Updated genome assembly of bighead carp (*Hypophthalmichthys nobilis*) provided insights into the evolution of sex determination systems in Cyprinidae

Running title: end-to-end genome assembly of bighead carp

Beide Fu[1], Ying Zhou[1,2], Haiyang Liu[3], Xiaomu Yu[1], Jingou Tong[1*]

1. State Key Laboratory of Freshwater Ecology and Biotechnology, Institute of Hydrobiology, The Innovation Academy of Seed Design, Chinese Academy of Sciences, Wuhan 430072, China
2. University of Chinese Academy of Sciences, Beijing 100039, China
3. Key Laboratory of Tropical and Subtropical Fishery Resources Application and Cultivation, Ministry of Agriculture and Rural Affairs, Pearl River Fisheries Research Institute, Chinese Academy of Fishery Sciences, Guangzhou 510380, China

Corresponding author: Jingou Tong,

Tel: +86-27-68780751, jgtong@ihb.ac.cn

Key words: end-to-end, genome assembly, bighead carp, methylation

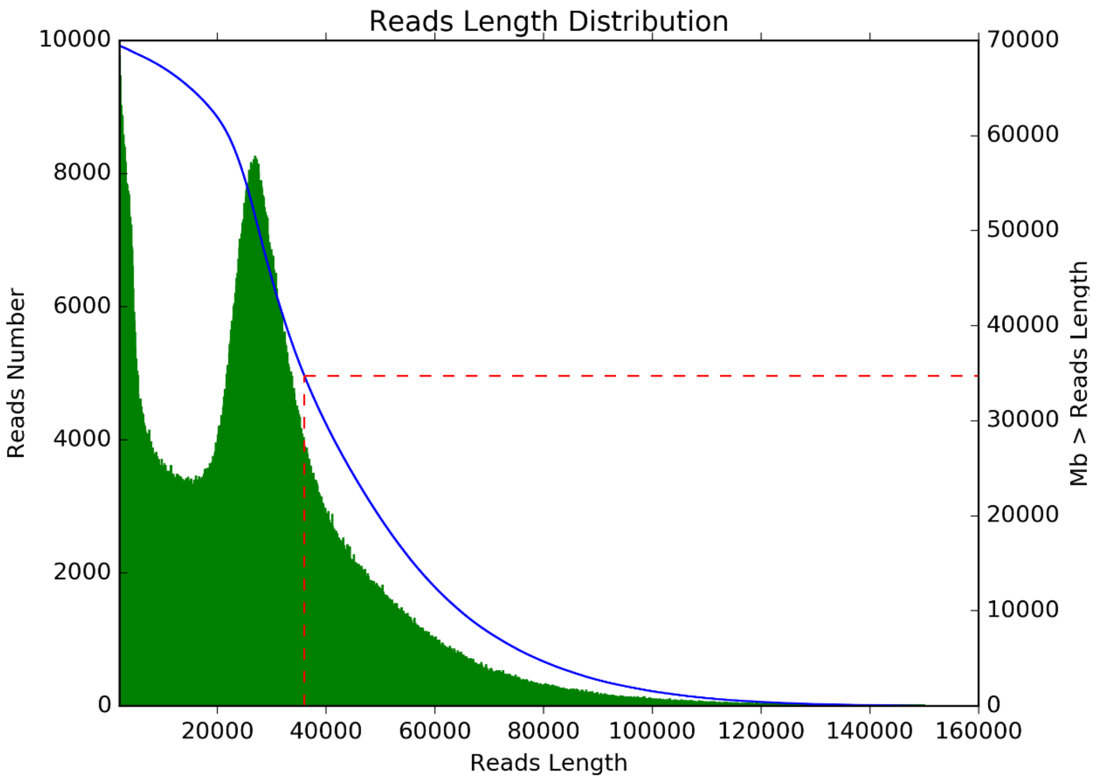

Supplementary Figure 1. Length distribution for filtered nanopore reads.

The green bar corresponding to the left ordinate (Reads number); The blue line corresponding to the right ordinate. The red dish line corresponding to the reads N50.

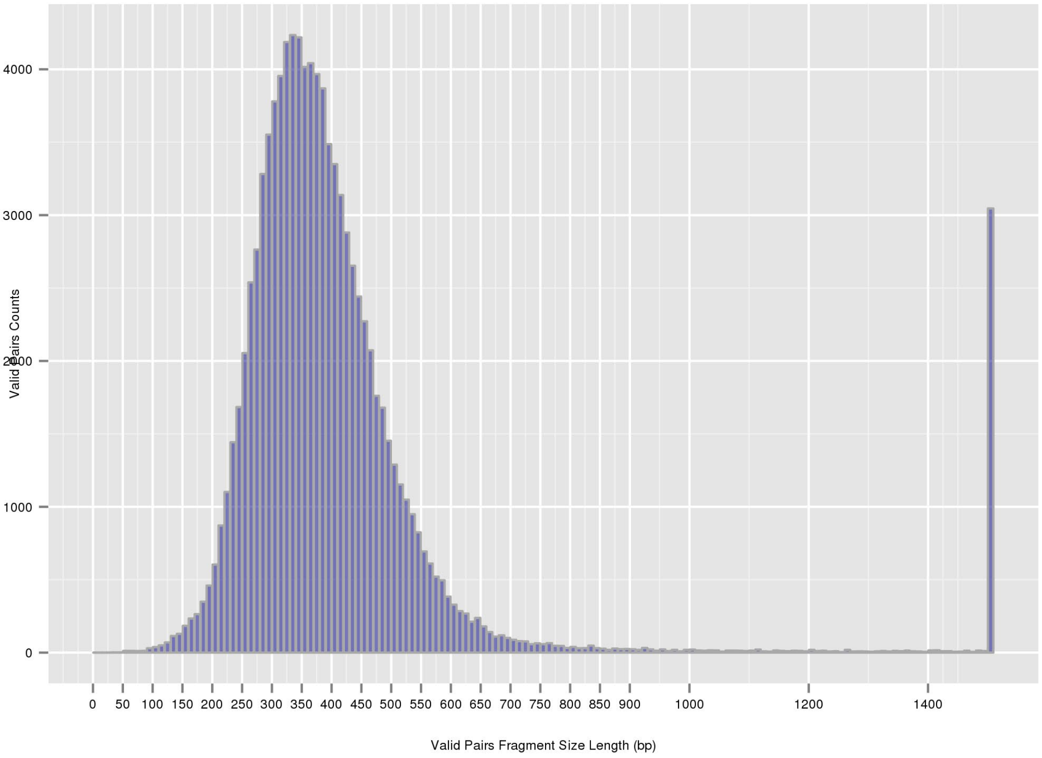

Supplementary Fig2. Fragment size of Hi-C library.

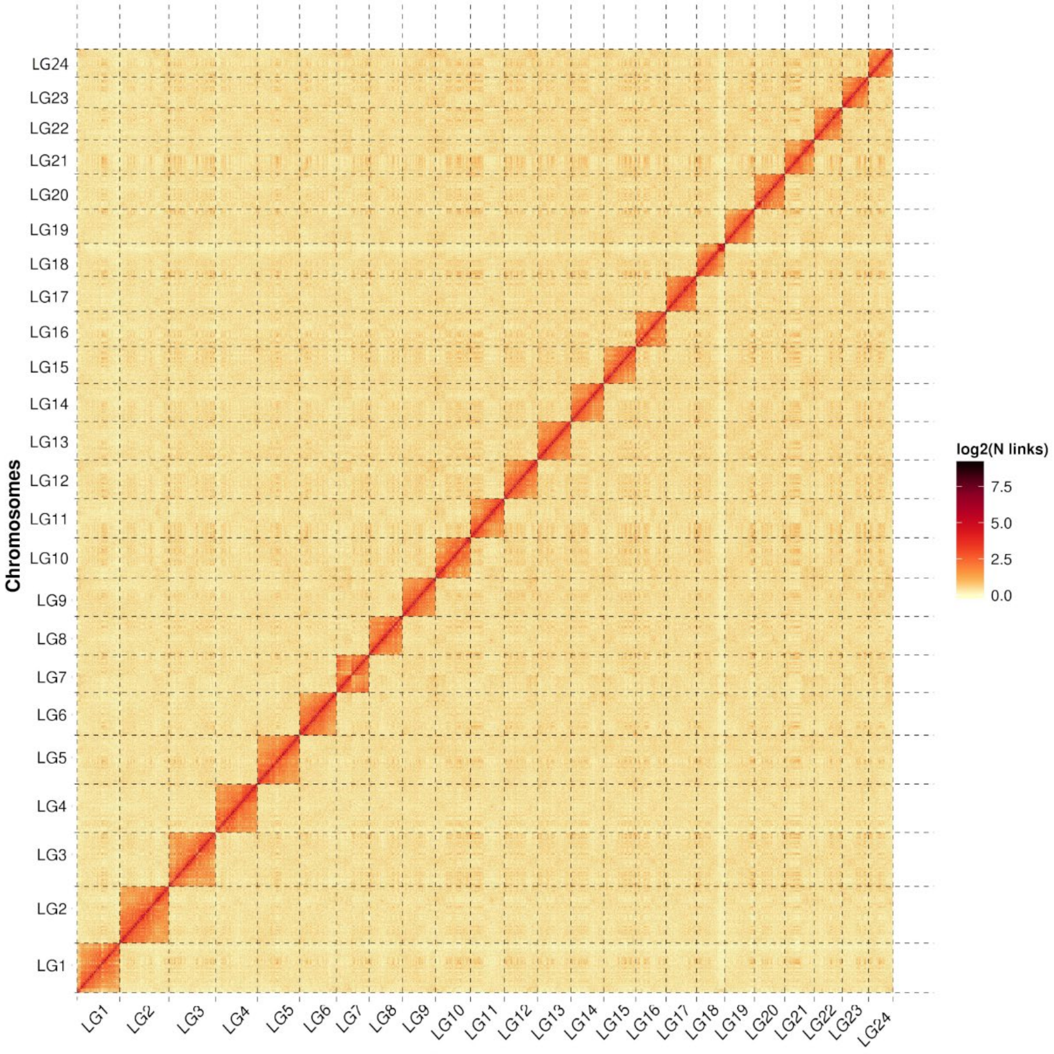

Supplementary Figure 3. Hi-C heatmap of bighead carp genome.

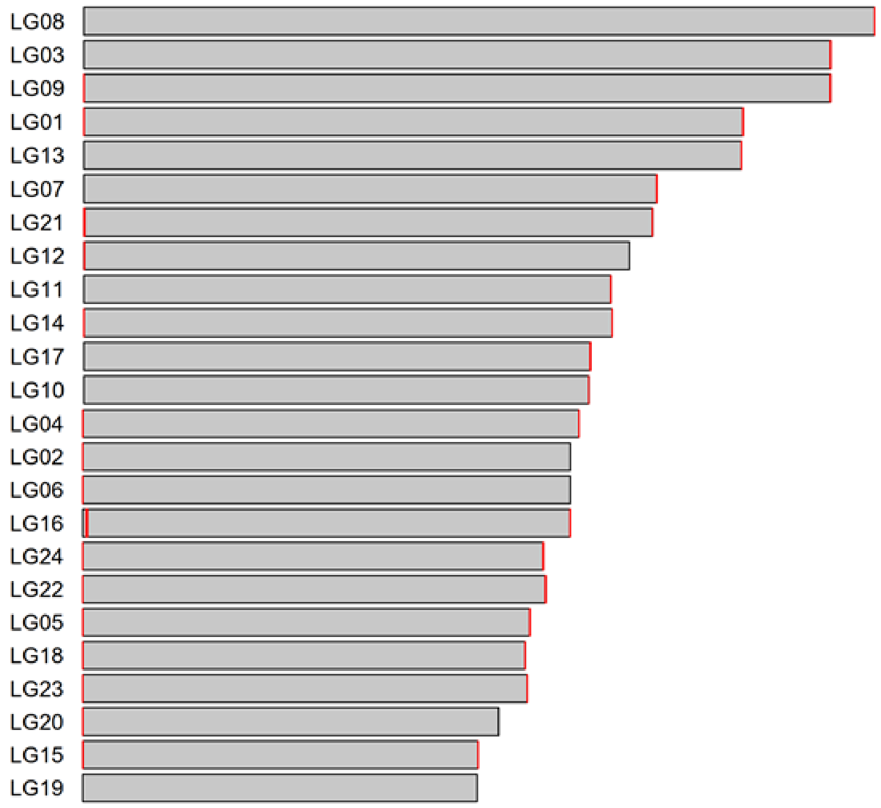

Supplementary Figure 4. Telomere position on each chromosome in bighead carp genome.

The red bar represents telomere region at the end of chromosome.

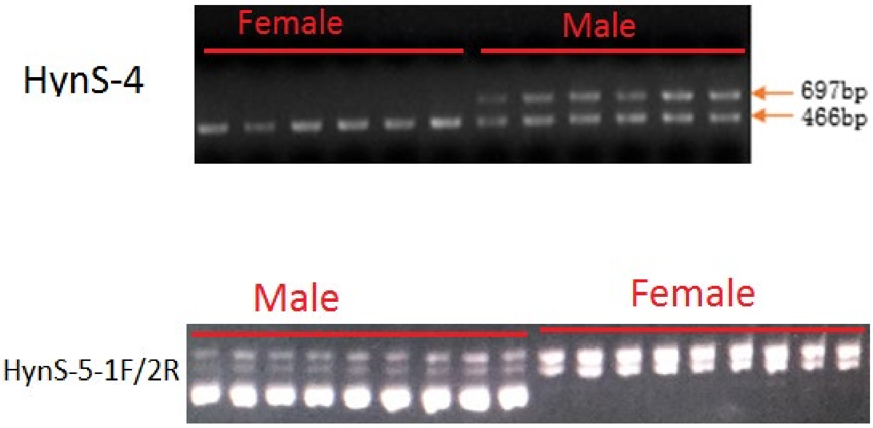

Supplementary Figure 5. Two markers were used to verify the authenticity of male-specific region on LG19.

The upper HynS-4 (697bp) came from Scaffold928 and the primer sequences is

HynS-4F:CCAATGGCGAGTTTCAC, HynS-4R:CGCAAGGCAGTGTTTCTA.

The reference in HynS-4 (466bp) with primer:

HynGapdh-3F:GGGAAGTTTGCGTATTTG, HynGapdh-3R:CAAAGATAGATGAGCGACAA

The lower part HynS-5 comes from Scaffold17972, and its primer sequences is

HynS-5-1F: TAGTTATTGCTTGGGTCA, HynS-5-2R: TACGCTTGTCTTAGTGAAA

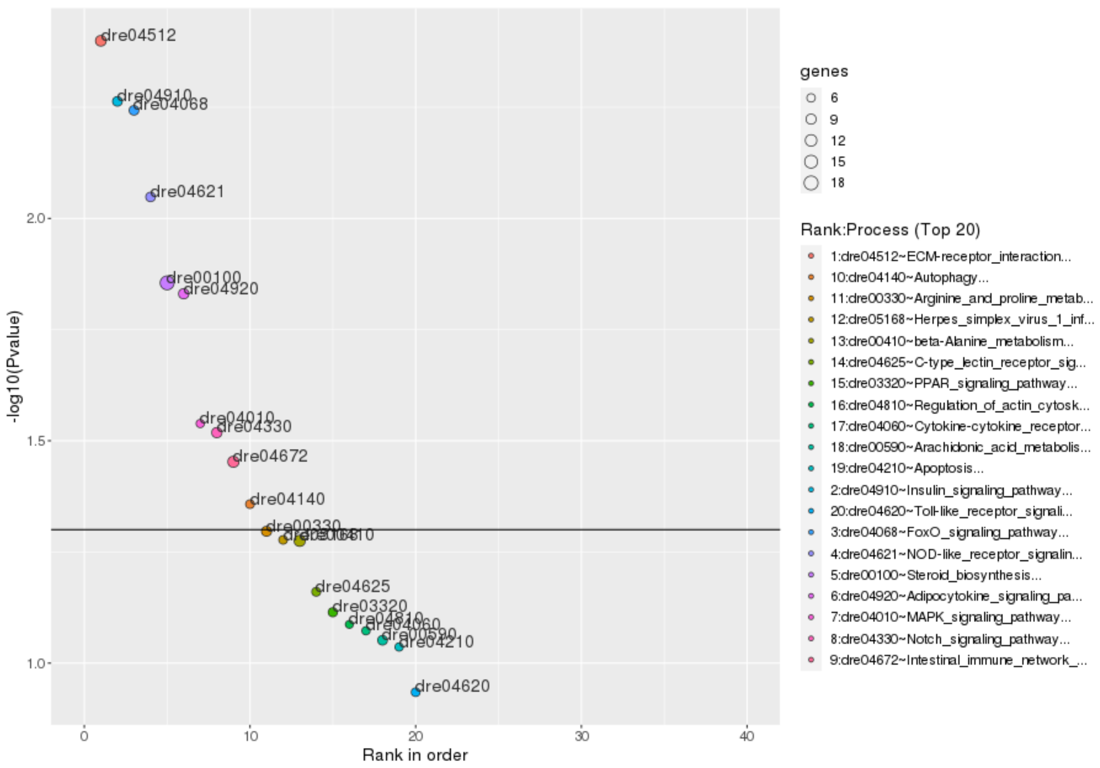

Supplementary Figure 6. GO enrichment analysis of 262 differentially expressed genes between male and female bighead carp’s hypothalamus transcriptome.

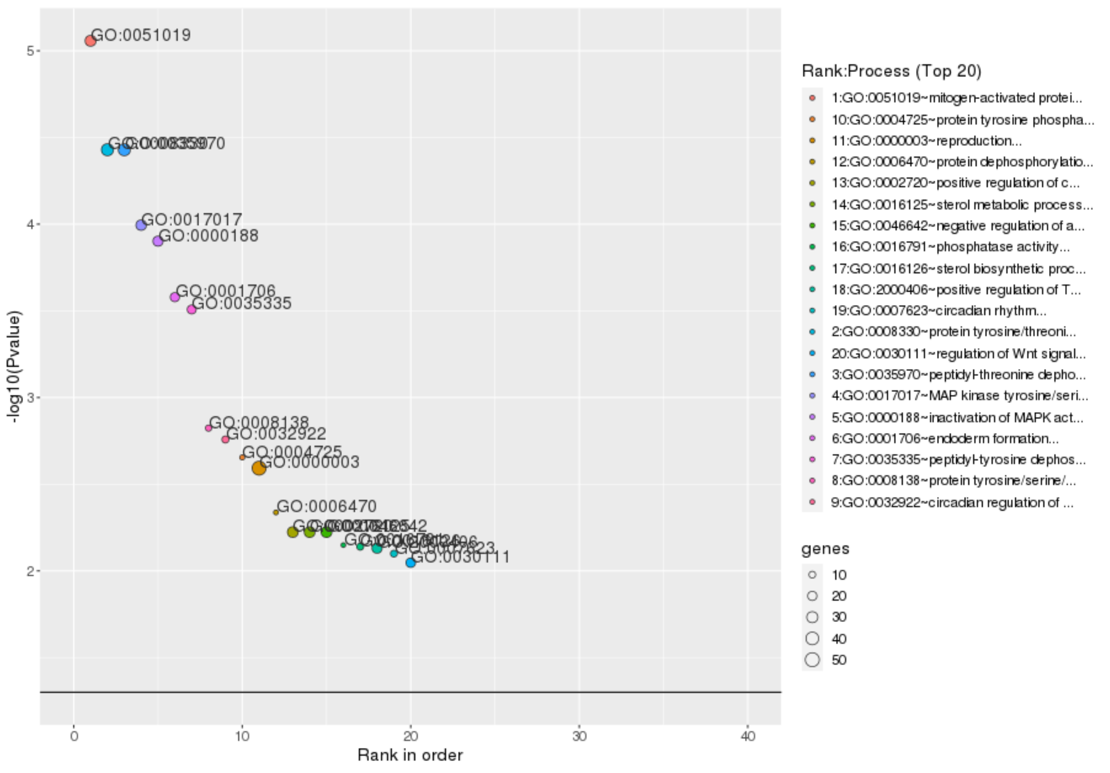

Supplementary Figure 7. GO enrichment analysis of 234 differentially expressed genes between male and female bighead carp's pituitary transcriptome.

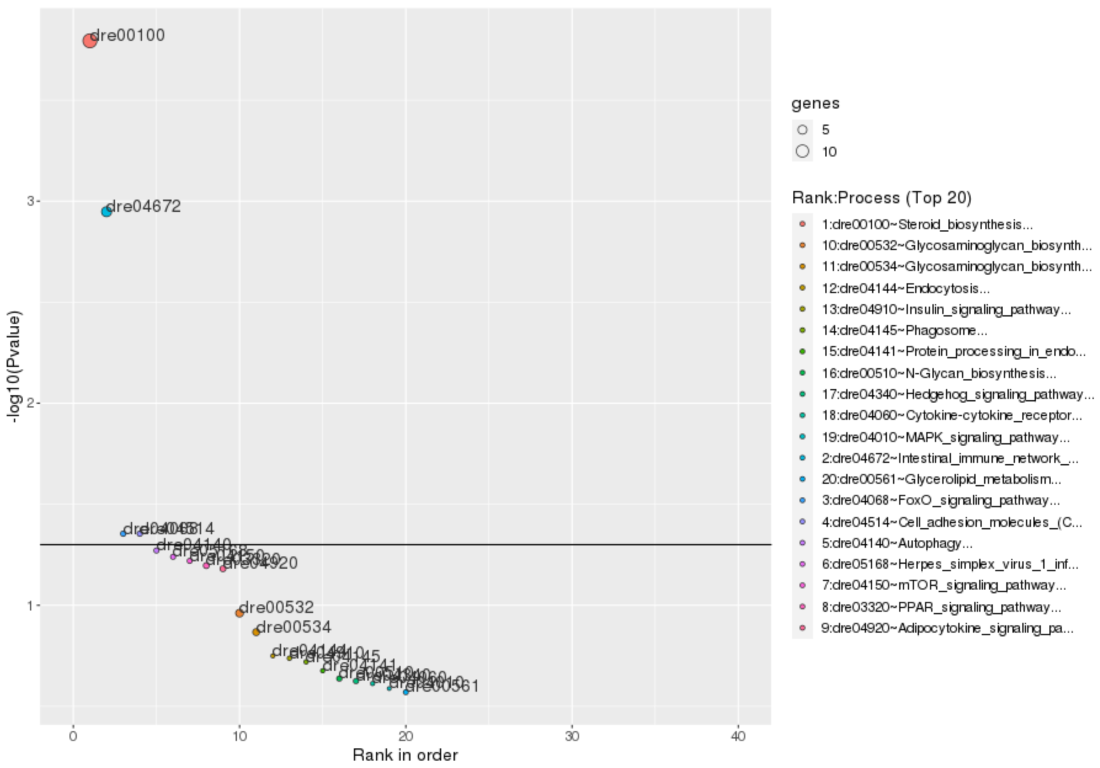

Supplementary Figure 8. KEGG enrichment analysis of 117 differentially expressed genes between male and female bighead carp’s hypothalamus and pituitary transcriptome.

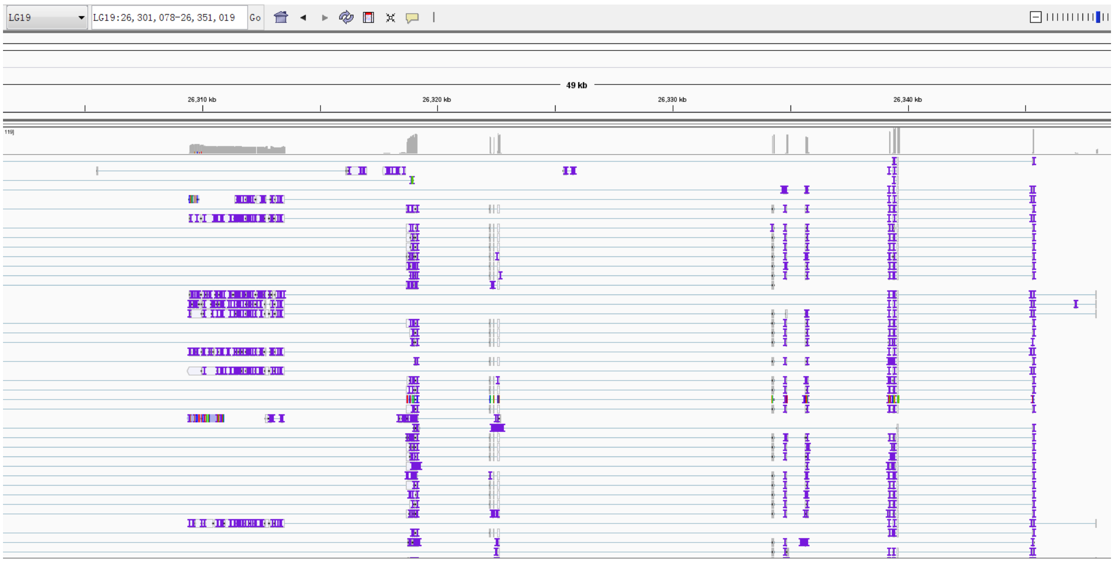

Supplementary Figure 9. Gene structure of one lncRNA on male-specific region of LG19.

This lncRNA has about 110X coverage in one ONT full-length brain transcriptome of a male bighead carp.
