# Supplementary material for: Updated Genome Assembly of Bighead Carp (Hypophthalmichthys nobilis) and Its Differences Between Male and Female on Genomic, Transcriptomic, and Methylation Level
Source: Front Genet. 2021 Sep 6;12:728177. doi: 10.3389/fgene.2021.728177 (PMC8452039; doi:10.3389/fgene.2021.728177)
Supplement: Supplementary file 6 [file Table_1.docx]

Updated genome assembly of bighead carp (*Hypophthalmichthys nobilis*) provided insights into the evolution of sex determination systems in Cyprinidae

Running title: end-to-end genome assembly of bighead carp

Beide Fu^1^, Ying Zhou^1,2^, Haiyang Liu^3^, Xiaomu Yu^1^, Jingou Tong^1*^

1. State Key Laboratory of Freshwater Ecology and Biotechnology, Institute of Hydrobiology, The Innovation Academy of Seed Design, Chinese Academy of Sciences, Wuhan 430072, China
2. University of Chinese Academy of Sciences, Beijing 100039, China
3. Key Laboratory of Tropical and Subtropical Fishery Resources Application and Cultivation, Ministry of Agriculture and Rural Affairs, Pearl River Fisheries Research Institute, Chinese Academy of Fishery Sciences, Guangzhou 510380, China

Corresponding author: Jingou Tong,

Tel: +86-27-68780751, [jgtong@ihb.ac.cn](mailto:jgtong@ihb.ac.cn)

Key words: end-to-end, genome assembly, bighead carp, methylation

Supplementary Table 1. Statistics for clean reads from Nanopore sequencing

| Seq Number | Sum of Base | N50 Length | N90 Length | Mean Length | Max Length | Mean Quality |
| --- | --- | --- | --- | --- | --- | --- |
| 2,412,655 | 69,589,433,624 | 36,094 | 19,304 | 28,843 | 417,624 | 8.33 |

Supplementary Table 2. Length distribution for filtered Nanopore reads.

| Length | Reads Number | Total Length (bp) | Percent of all bases | Average Length (bp) |
| --- | --- | --- | --- | --- |
| 2,000~5,000 | 230,168 | 776,675,622 | 1.11% | 3,374.38 |
| 5,000~10,000 | 203,711 | 1,498,116,751 | 2.15% | 7,354.12 |
| 10,000~20,000 | 345,961 | 5,205,674,322 | 7.48% | 15,046.99 |
| 20,000~30,000 | 663,802 | 16,913,651,185 | 24.30% | 25,479.96 |
| 30,000~40,000 | 448,301 | 15,367,474,574 | 22.08% | 34,279.36 |
| 40,000~50,000 | 222,217 | 9,905,007,263 | 14.23% | 44,573.58 |
| 50,000~60,000 | 133,115 | 7,260,637,989 | 10.43% | 54,544.10 |
| 60,000~70,000 | 74,491 | 4,803,579,223 | 6.90% | 64,485.36 |
| 70,000~80,000 | 40,845 | 3,043,807,563 | 4.37% | 74,520.93 |
| >=80,000 | 50,044 | 4,814,809,132 | 6.91% | 96,211.51 |

Supplementary Table 3. Hi-C reads alignment results.

| Alignment Type | Alignment Number | Ratio (%) |
| --- | --- | --- |
| Unique Paired Alignments | 167,809,907 | 100 |
| Valid Interaction Pairs | 119,333,569 | 71.11 |
| Dangling End Pairs | 28,679,044 | 17.09 |
| Re-ligation Pairs | 3,841,416 | 2.29 |
| Self-cycle Pairs | 892,773 | 0.53 |
| Dumped Pairs | 15,063,105 | 8.98 |

Supplementary Table 4. Estimation of genome size of the rubber tree using K-mer

| kmer | Kmer num | kmer  depth | Genome  size (Mb) | Used base | Used read | X |
| --- | --- | --- | --- | --- | --- | --- |
| 17 | 35384843826 | 40 | 884.621 | 40024115810 | 289954499 | 45.24 |

Supplementary Table 5. The length distribution of 24 LG in bighead carp genome.

| LG Name | Length |
| --- | --- |
| LG01 | 43885911 |
| LG02 | 32927637 |
| LG03 | 49710932 |
| LG04 | 33516304 |
| LG05 | 30203557 |
| LG06 | 32927357 |
| LG07 | 38144965 |
| LG08 | 52635676 |
| LG09 | 49695232 |
| LG10 | 33618591 |
| LG11 | 35072195 |
| LG12 | 36313995 |
| LG13 | 43780834 |
| LG14 | 35163629 |
| LG15 | 26702626 |
| LG16 | 32918314 |
| LG17 | 33747976 |
| LG18 | 29888619 |
| LG19 | 26635474 |
| LG20 | 28094456 |
| LG21 | 37858798 |
| LG22 | 31289655 |
| LG23 | 30033327 |
| LG24 | 31146994 |

Supplementary Table 6. Telomere sequence positions on 24 chromosomes.

| Chr | Start | End |
| --- | --- | --- |
| LG01 | 0 | 9001 |
| LG01 | 9002 | 14100 |
| LG01 | 14101 | 43859471 |
| LG01 | 43859472 | 43885911 |
| LG02 | 5 | 11876 |
| LG03 | 49685850 | 49710921 |
| LG04 | 328 | 25061 |
| LG04 | 33510280 | 33516301 |
| LG05 | 1 | 20330 |
| LG05 | 30195580 | 30203554 |
| LG06 | 527 | 17916 |
| LG07 | 38129199 | 38144965 |
| LG08 | 52623591 | 52635673 |
| LG09 | 7476 | 15111 |
| LG09 | 49685158 | 49695229 |
| LG10 | 33599131 | 33599221 |
| LG11 | 35062282 | 35072189 |
| LG12 | 20390 | 48781 |
| LG13 | 43764528 | 43780830 |
| LG14 | 501 | 15375 |
| LG14 | 35149845 | 35163431 |
| LG15 | 279 | 35360 |
| LG15 | 26696478 | 26702582 |
| LG16 | 236519 | 322115 |
| LG16 | 32903869 | 32918314 |
| LG17 | 33687339 | 33747955 |
| LG18 | 7594 | 18560 |
| LG18 | 29880171 | 29881169 |
| LG20 | 6 | 19377 |
| LG21 | 132 | 70098 |
| LG21 | 37839033 | 37848929 |
| LG22 | 266 | 8733 |
| LG22 | 31243596 | 31288810 |
| LG23 | 7837 | 8657 |
| LG23 | 30022770 | 30033320 |
| LG24 | 1 | 11230 |
| LG24 | 31082283 | 31144019 |

Supplementary Table 7. Align of pair end Illumina reads to assembled bighead carp genome.

| Align Type | Align num | Ratio (%) |
| --- | --- | --- |
| concordantly 0 times | 8798380 | 6.07 |
| concordantly exactly 1 time | 111984473 | 77.24 |
| concordantly >1 time | 24203133 | 16.69 |
| Total | 144985986 | 100 |

Supplementary Table 8. BUSCO evaluation for the assembled bighead carp genome with Actinopterygii_odb9.

| Type | Total gene num | Mapped gene num | Ratio (%) |
| --- | --- | --- | --- |
| Complete BUSCOs (C) | 4584 | 4363 | 95.2 |
| Complete and single-copy BUSCOs (S) | 4584 | 4200 | 91.6 |
| Complete and duplicated BUSCOs (D) | 4584 | 163 | 3.6 |
| Fragmented BUSCOs (F) | 4584 | 76 | 1.7 |
| Missing BUSCOs (M) | 4584 | 145 | 3.1 |

Supplementary Table 9. Statistics of repeat sequences in the bighead carp genome.

| Type | number of elements | length occupied (bp) | percentage of sequence (%) |
| --- | --- | --- | --- |
| SINEs: | 30278 | 3058481 | 0.36 |
| ALUs | 0 | 0 | 0 |
| MIRs | 124 | 6315 | 0 |
| LINEs: | 47026 | 19292586 | 2.25 |
| LINE1 | 6008 | 1898850 | 0.22 |
| LINE2 | 24363 | 11660288 | 1.36 |
| L3/CR1 | 0 | 0 | 0 |
| LTR elements: | 146201 | 53848251 | 6.28 |
| ERVL | 0 | 0 | 0 |
| ERVL-MaLRs | 0 | 0 | 0 |
| ERV_classI | 8467 | 1615422 | 0.19 |
| ERV_classII | 0 | 0 | 0 |
| DNA elements: | 1655436 | 282745909 | 32.99 |
| hAT-Charlie | 18620 | 1532009 | 0.18 |
| TcMar-Tigger | 94 | 3444 | 0 |
| Unclassified: | 399753 | 55296596 | 6.45 |
|  |  |  |  |
| Total Interspersed repeats: |  | 414241823 | 48.33 |
| Small RNA: | 0 | 0 | 0 |
| Satellites: | 6154 | 1121789 | 0.13 |
| Simple repeats: | 89482 | 15571258 | 1.82 |
| Low complexity: | 413 | 95876 | 0.01 |

Supplementary Table 16. Depth statistics for Nanopore reads’ 5-mC methylation

| Percentage in the whole dataset (%) | Depth |
| --- | --- |
| 0 | 1 |
| 10 | 6 |
| 20 | 8 |
| 30 | 10 |
| 40 | 11 |
| 50 | 13 |
| 60 | 14 |
| 70 | 16 |
| 80 | 20 |
| 90 | 26 |
| 100 | 1449 |
